# Neuron-Specific Feeding RNAi in *C. elegans* and Its Use in a Screen for Essential Genes Required for GABA Neuron Function

**DOI:** 10.1371/journal.pgen.1003921

**Published:** 2013-11-07

**Authors:** Christopher Firnhaber, Marc Hammarlund

**Affiliations:** Department of Genetics, Program in Cellular Neuroscience, Neurodegeneration, and Repair, Yale University School of Medicine, New Haven, Connecticut, United States of America; Stanford University School of Medicine, United States of America

## Abstract

Forward genetic screens are important tools for exploring the genetic requirements for neuronal function. However, conventional forward screens often have difficulty identifying genes whose relevant functions are masked by pleiotropy. In particular, if loss of gene function results in sterility, lethality, or other severe pleiotropy, neuronal-specific functions cannot be readily analyzed. Here we describe a method in *C. elegans* for generating cell-specific knockdown in neurons using feeding RNAi and its application in a screen for the role of essential genes in GABAergic neurons. We combine manipulations that increase the sensitivity of select neurons to RNAi with manipulations that block RNAi in other cells. We produce animal strains in which feeding RNAi results in restricted gene knockdown in either GABA-, acetylcholine-, dopamine-, or glutamate-releasing neurons. In these strains, we observe neuron cell-type specific behavioral changes when we knock down genes required for these neurons to function, including genes encoding the basal neurotransmission machinery. These reagents enable high-throughput, cell-specific knockdown in the nervous system, facilitating rapid dissection of the site of gene action and screening for neuronal functions of essential genes. Using the GABA-specific RNAi strain, we screened 1,320 RNAi clones targeting essential genes on chromosomes I, II, and III for their effect on GABA neuron function. We identified 48 genes whose GABA cell-specific knockdown resulted in reduced GABA motor output. This screen extends our understanding of the genetic requirements for continued neuronal function in a mature organism.

## Author Summary

Living organisms often reuse the same genes multiple times for different purposes. If one function of a gene is essential, death or arrest of the mutant masks other functions. Understanding the functions of essential genes is particularly critical in the nervous system, which must maintain plasticity and fend off disease long after development is complete. However, current strategies for generating conditional knockouts rely on making a new transgenic animal for each gene and thus are not useful for forward genetic screens or for other experiments involving a large number of genes. We have developed a technique in *C. elegans* for generating gene knockdown in selected neuron sub-types in response to feeding RNAi. Using this technique, we performed a screen aimed at identifying essential genes that are required for the function of mature GABAergic neurons. By knocking these genes down in only GABAergic neurons, we can circumvent the muddying effects of pleiotropy and find essential genes that function cell intrinsically to promote GABA neuron function. The genes we identified using this method provide a more complete understanding of the complex genetic requirements of post-developmental neurons.

## Introduction

In *C. elegans*, there are two basic ways to generate mosaic gene expression: knocking gene function down in specific cells of an otherwise normal animal; or rescuing wild type gene function in a mutant animal. Examples of the first method include triggering local RNAi by targeted expression of hairpin or double-stranded RNA [1], [2]; examples of the second include the use of unstable DNA elements or the targeted expression of wildtype coding sequences [3]. However, all of these methods require the construction of a new transgenic animal for each gene of interest. The requirement for one strain per gene limits the usefulness of these techniques for questions involving many genes, because it is impractical to construct so many transgenic animals.

Gene knockdown in *C. elegans* can be induced by feeding RNAi [4], and the development of whole-genome feeding RNAi libraries means that RNAi can be used for large-scale genetic analysis, up to and including whole-genome screens [5]–[7]. Moreover, many of the cellular mechanisms that mediate feeding RNAi have been described. In particular, interfering RNA species enter cells using the dsRNA channel SID-1 [8]–[10], while within each cell the Argonaute protein RDE-1 is required to achieve gene knockdown [11]. This molecular understanding has been used to develop a new method for mosaic gene expression that is generated by feeding RNAi, and thus is compatible with the study of many genes. For example, a muscle-specific *rde-1* mosaic enables muscle-specific knockdown in response to feeding RNAi [12]. Similarly, manipulating *sid-1* expression can alter the response of touch neurons to feeding RNAi [13].

Neurons, however, present particular problems for feeding RNAi. Most *C. elegans* neurons are resistant to feeding RNAi [14]–[16]. Genetic backgrounds have been developed that enhance the sensitivity of neurons to feeding RNAi, such as the *lin-15B; eri-1* mutant [17] and neuronal expression of *sid-1* [13]. However, these same genetic backgrounds can also result in increased transgene silencing in the nervous system [17]–[19]. Such transgene silencing could limit expression of the transgenes driving mosaic rescue of RNAi, even while these mutations enhance RNAi sensitivity. Thus, an approach that allows feeding RNAi to generate tissue-specific gene knockdown in neurons that can be generalized to a variety of neuronal subtypes is not currently available. Here, we describe a strategy in *C. elegans* that allows feeding RNAi to generate cell-specific knockdown in a wide variety of neuronal subtypes. We use this method to examine the genetic requirements of mature GABA motor neurons.

## Results

### An approach to neuron-specific feeding RNAi

**Figure 1:**
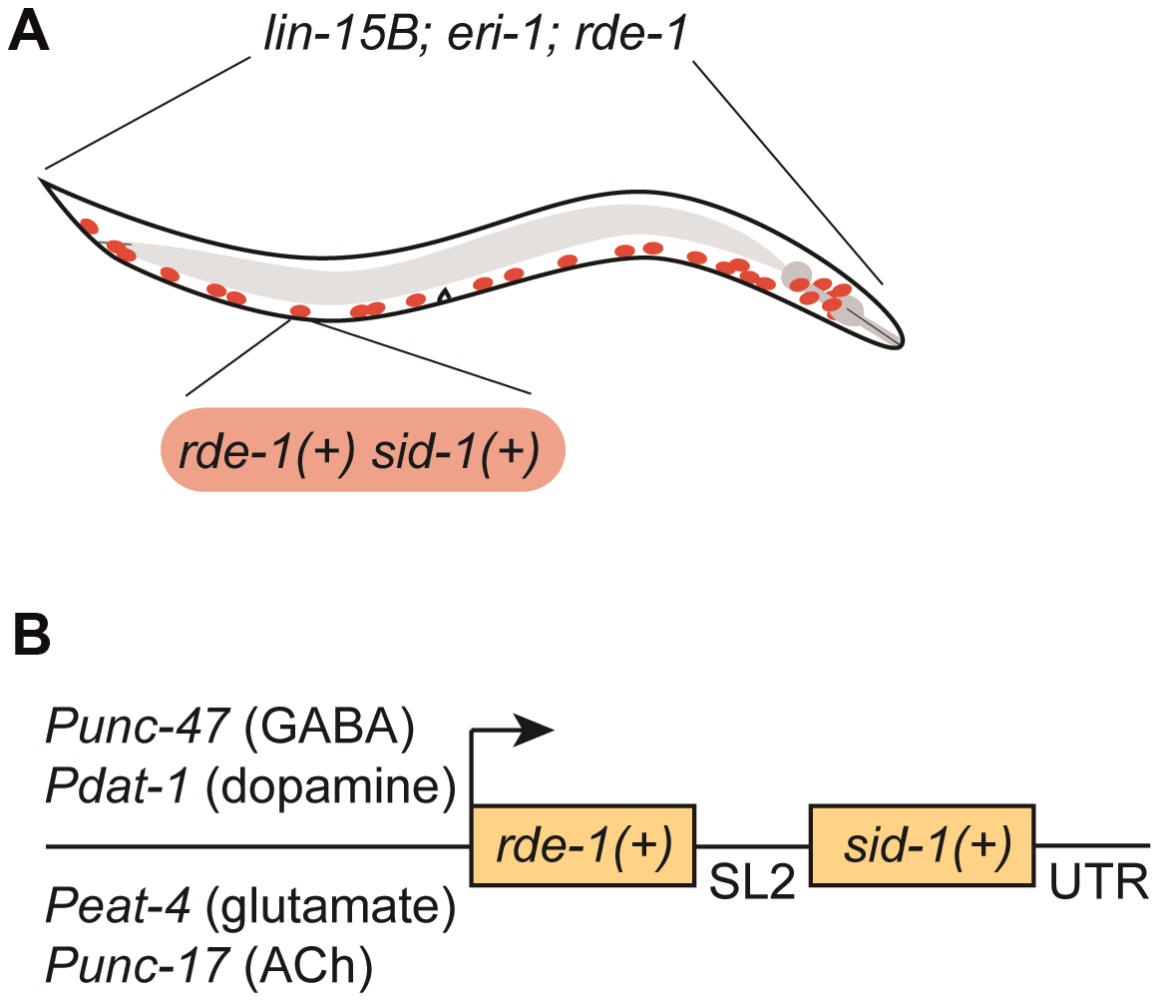
A feeding RNAi-compatible approach to neuron-specific knockdown. (**A**) Animals carry a genetic background of *lin-15B(n744); eri-1(mg366); rde-1(ne219)*, and express wildtype *sid-1(+)* and *rde-1(+)* in selected neurons (GABA neurons are depicted). (**B**) Wildtype *sid-1* and *rde-1* are expressed from an artificial operon under various neuron sub-type-specific promoters and inserted as a single copy into the genome by MosSCI.

**Figure 2:**
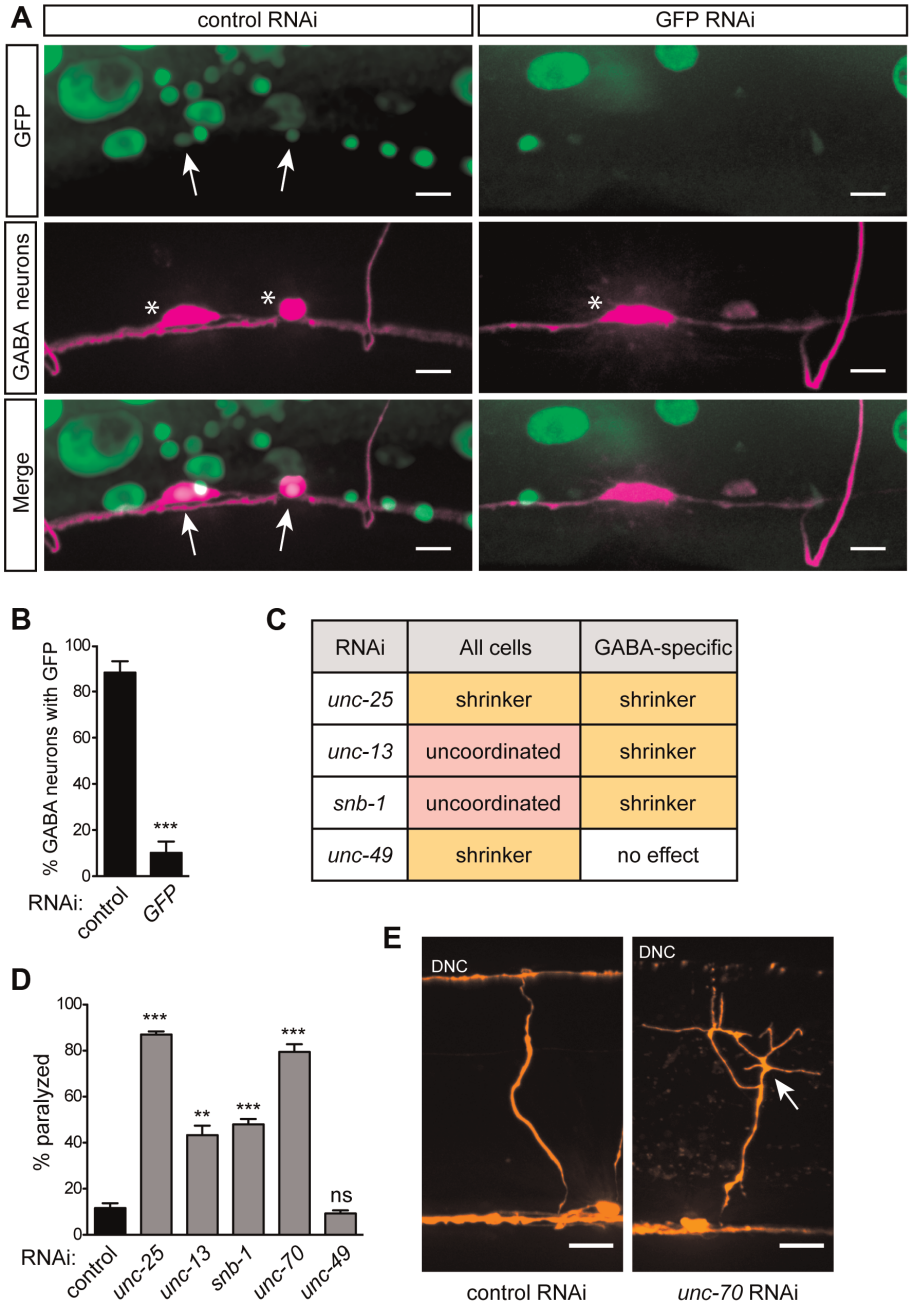
GABA-specific RNAi. (**A**) P*unc-47* (GABA-specific) RNAi strain expressing ubiquitous, nuclear-localized GFP and GABA mCherry after control or *GFP* RNAi feeding. Arrows mark GABA neurons with nuclear GFP. Stars mark GABA neuron cell bodies. *GFP* RNAi eliminates GFP in GABA cells, while GFP remains in other cell types. In control RNAi animals, GFP in GABA neurons is reduced relative to other cells due to increased transgene silencing in these neurons (see text). Scale bars = 5 µm. (**B**) Quantification of the percent of GABA cell bodies with nuclear-localized GFP under control or *GFP* RNAi conditions. Error bars are SEM, n = 10 worms, *** p<0.0001 (**C**) Locomotion behaviors in response to RNAi against genes known to function in GABA neurotransmission, in animals sensitive to RNAi in all cells (*lin-15B; eri-1*) and in the GABA-specific strain (XE1375). In GABA-specific RNAi strain, a GABA-specific shrinker phenotype is observed when pre- but not post-synaptic GABA signaling genes are targeted. (**D**) Percent of paralyzed animals after 100 min of acute exposure to 750 µM aldicarb. Error bars are SEM, n = 3 trials of ∼25 animals each, ** p = 0.0023, *** p≤0.0003. (**E**) GABA commissures after control or *unc-70* RNAi. In *unc-70* RNAi, the axon has broken and initiated a regenerative growth cone (arrow), and the dorsal nerve cord (DNC) has degenerated. Scale bars = 10 µm.

We chose to restrict RNAi sensitivity to selected neurons using *rde-1* mosaic animals [12]. At the same time, we also sought to increase RNAi sensitivity in the selected neurons, since many neurons are resistant to RNAi. We used two complementary techniques to increase neuronal sensitivity. First, we used a genetic background (*lin-15B; eri-1*) that enhances the sensitivity of all neurons to RNAi [17]. Second, we overexpressed the double-stranded RNA transporter *sid-1* only in the selected neurons [13]. Thus, our strain carries three background mutations (*lin-15B; eri-1; rde-1*) and expresses two transgenes in select neurons (*rde-1(+)* and *sid-1(+)*) (Fig. 1A). We initially found that our approach was subject to significant transgene silencing effects, likely caused by the combination of the *lin-15B; eri-1* background and *rde-1* overexpression (see Fig. 2A) [17], [18]. To avoid transgene silencing, we combined the *rde-1(+)* and *sid-1(+)* rescue fragments into a single transcriptional unit, separated by an SL2-specific trans-splice site [20]. This artificial operon was placed in a MosSCI-compatible [21] MultiSite Gateway vector for easy manipulation, single-copy integration, and expression under cell-specific promoters.

### GABAergic neuron-specific RNAi

We first targeted GABA-releasing neurons. We used Gateway recombination to place the *rde-1(+)*; *sid-1(+)* operon behind the P*unc-47* promoter, which drives expression exclusively in the GABA motor neurons (Fig. 1B) [22]. This construct was inserted into the genome as a single copy using the MosSCI technique [21], and this transgene was crossed into the *lin-15B; eri-1; rde-1* mutant background to generate a strain of animals in which the interfering response of exogenous dsRNA is limited to GABA neurons.

To determine the effectiveness of our approach, we expressed nuclear-localized GFP in all somatic cells of our GABA-specific RNAi strain and also marked the GABA neurons with mCherry. We fed these animals RNAi against GFP and found that, compared to control RNAi, GFP was efficiently knocked down in GABA neurons but was still present in other cells – including muscles, intestine, skin, and non-GABA neurons (Fig. 2A, B). This suggests that the RNAi response is limited to the tissue in which our artificial operon is expressed and does not spread to other adjacent cells or tissues.

We also tested the viability of our strain when challenged with RNAi against an essential gene. *ama-1* encodes the large subunit of *C. elegans* RNA polymerase II [23], and RNAi against *ama-1* results in a larval arrest phenotype with penetrance approaching 100%. By contrast, the GABA-specific strain, and the others described below, were completely resistant to *ama-1* RNAi-induced arrest, demonstrating that gene function can be studied in the GABA neurons of an otherwise normal animal even when these genes have essential functions in other tissues.

Next, we sought to determine the effectiveness of our approach against endogenous, single-gene targets. We took advantage of the robust and specific behavioral ‘shrinker’ phenotype generated by lack of either pre- or post-synaptic components of GABA neurotransmission. The GABA-specific shrinker phenotype is readily distinguished from the paralyzed phenotype caused by loss of basal neurotransmission components. We compared the effect of feeding RNAi between a standard neuron-sensitized strain (*lin-15B; eri-1*) and our GABA-specific strain. We performed RNAi against two GABA-specific genes: *unc-25*, which encodes glutamic acid decarboxylase [24] and is required in GABA neurons for GABA neurotransmission; and *unc-49*, which encodes the GABA_A_ receptor [25] and is required in muscles for GABA neurotransmission. We also targeted two components of basal neurotransmission, both of which are required in all neurons for synaptic vesicle release: *unc-13*, which encodes UNC-13 [26], and *snb-1*, which encodes synaptobrevin [27]. We found that, as expected, knockdown of GABA genes in the neuron-sensitized strain resulted in a GABA-specific shrinker phenotype, while knockdown of basal neurotransmission genes resulted in an uncoordinated phenotype. By contrast, in our GABA-specific strain, knockdown of the neuronal GABA gene *unc-25* resulted in a shrinker phenotype, while knockdown of the muscle GABA gene *unc-49* had no effect. Further, in our GABA-specific strain, knockdown of basal neurotransmission genes also resulted in a shrinker phenotype (Fig. 2C). To quantify behavioral changes due to GABA-specific knockdown, we utilized an aldicarb-sensitivity assay to indirectly measure GABA output. Aldicarb is an acetylcholinesterase inhibitor that causes acute paralysis due to accumulation of acetylcholine at neuromuscular junctions (NMJs). Loss of inhibitory GABA input leads to hypersensitivity to aldicarb, causing more rapid paralysis [28]. As expected, GABA-specific knockdown of *unc-25* as well as the basal neurotransmission genes *unc-13* and *snb-1* led to hypersensitivity to aldicarb, while RNAi against *unc-49* had no effect (Fig. 2D).

In addition to synaptic genes, we sought to target a broadly-expressed gene involved in maintenance of the nervous system. *unc-70* encodes β-spectrin, a component of the plasma membrane skeleton that is expressed in all cells [29]. Animals lacking *unc-70* generate spontaneous breaks in their neurons as a result of mechanical stress [30]. Neuron-sensitized (*lin-15B; eri-1*) animals fed dsRNA against *unc-70* are slow to grow, dumpy, and paralyzed. Knockdown of *unc-70* in the GABA-specific strain, however, caused an aldicarb hypersensitivity phenotype (Fig. 2D), with no other obvious phenotypes. When the GABA neurons of these worms were examined, we observed defects consistent with lack of *unc-70*, including branched processes, broken axons – some of which terminated in regenerative growth cones – as well as substantial degeneration of the dorsal nerve cord, especially where disconnection of distal fragments was apparent (Fig. 2E). Together, these data demonstrate that our approach allows knockdown of endogenous genes within selected neurons, while preventing knockdown of those genes in other cells. Moreover, this method allows for dissection of the site of gene action, easily separating pre- and post-synaptic functions, as well as neuron sub-type-specific effects of gene knockdown.

### Other neuron sub-types

To determine whether our system for controlling feeding RNAi was adaptable to other sets of neurons, we used other promoters to drive expression of our artificial *rde-1(+); sid-1(+)* operon, made single-copy MosSCI integrations of each construct, and placed each resulting MosSCI transgene in the *lin-15B; eri-1; rde-1* mutant background. Next, we characterized the resulting strains by challenging them with *ama-1* RNAi. Three of these new strains – those using the P*dat-1*, P*unc-17*, and P*eat-4* promoters – satisfied the test of *ama-1* RNAi resistance in this context, and further experiments with these three strains are discussed below. However, with two other promoters – P*rab-3* and P*mig-13* – we found that the resulting strain was not resistant to *ama-1* RNAi. Thus, these promoters are not suitable for the analysis of essential genes using our system. By contrast, the P*dat-1*, P*unc-17*, and P*eat-4* promoters appear to be tightly controlled, suggesting that the three strains using these promoters can be used for neuron-specific RNAi.

### Dopaminergic neuron-specific RNAi

**Figure 3:**
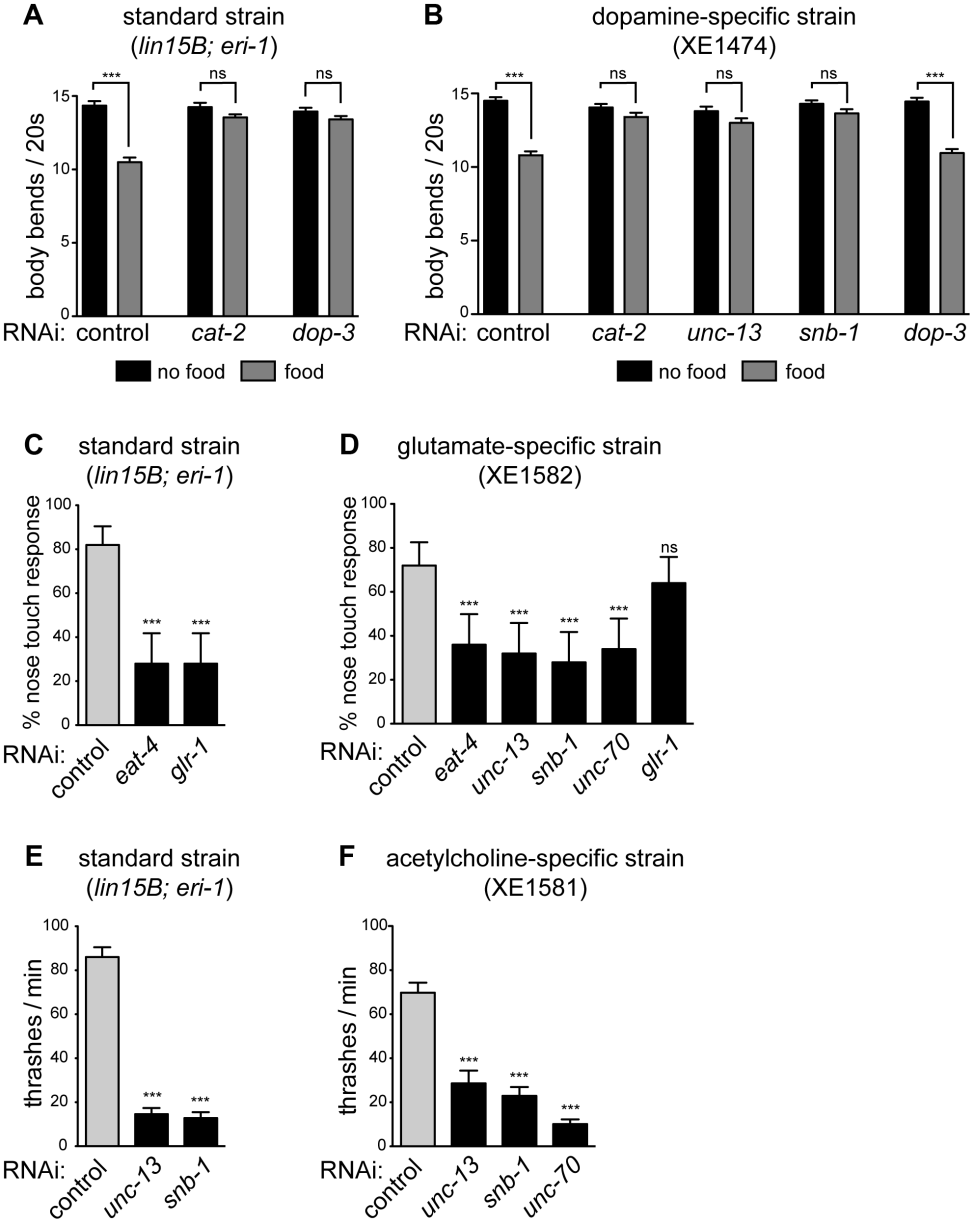
Specific gene knockdown in dopamine, glutamate, and acetylcholine neurons in response to feeding RNAi. (**A**) In standard neuron-sensitive strain, RNAi of presynaptic (*cat-2*) or postsynaptic (*dop-3*) components of dopamine signaling eliminates basal slowing on a bacterial lawn (gray bars). Error bars are SEM, n = 20 worms, *** p<0.0001. (**B**) In dopamine-specific strain, RNAi of a presynaptic component of dopamine signaling (*cat-2*) eliminates basal slowing, while RNAi of a postsynaptic component (*dop-3*) does not (gray bars). RNAi of presynaptic components of general neurotransmission (*unc-13* and *snb-1*) also eliminates basal slowing. Error bars are SEM, n = 20 worms, *** p<0.0001. (**C**) In standard neuron-sensitive strain, RNAi of presynaptic (*eat-4*) or postsynaptic (*glr-1*) components of glutamate signaling diminish the response to nose touch. Error bars are 95% confidence interval, n = 5 trials/animal, 10 animals, *** p<0.0001. (**D**) In glutamate-specific strain, RNAi of presynaptic components of glutamate neurotransmission (*eat-4, unc-13, snb-1*) as well as the structural protein *unc-70* inhibit the nose touch response, while RNAi of a postsynaptic component (*glr-1*) does not. Error bars are 95% confidence interval, n = 5 trials/animal, 10 animals, *** p≤0.0006. (**E**) In standard neuron-sensitive strain, RNAi of presynaptic components of general neurotransmission (*unc-13* and *snb-1*) slows rate of thrashing. Error bars are SEM, n = 10 worms, *** p<0.0001 (**F**) In acetylcholine-specific strain, RNAi of presynaptic components of general neurotransmission (*unc-13* and *snb-1*) as well as *unc-70* slows rate of thrashing. Error bars are SEM, n = 10 worms, *** p<0.0001.

P*dat-1* drives expression in the dopaminergic neurons [31], which comprise eight cells in adult hermaphrodites [32]. One function of dopamine release from these cells is to control a behavioral response to food called “basal slowing,” in which animals slow their rate of locomotion when they encounter a bacterial lawn [33]. We used a basal slowing assay to evaluate the ability of our P*dat-1* strain to restrict RNAi to the dopaminergic neurons. The *cat-2* gene encodes tyrosine hydroxylase and is required for dopamine synthesis [34]. Mutant animals that lack *cat-2*, or animals in which all eight dopamine neurons have been ablated, do not exhibit basal slowing [33]. Basal slowing response is also absent in mutants that lack *dop-3*, which encodes a D2 dopamine receptor and is not expressed in the dopaminergic neurons [35]. Thus, basal slowing requires factors both intrinsic and extrinsic to the dopamine neurons. We found that both a standard sensitized RNAi strain (*lin15B; eri-1*) and the P*dat-1* strain exhibited a robust basal slowing response on control RNAi (Fig. 3A, B). Further, the basal slowing response was completely blocked in both strains on RNAi against *cat-2*, which is required in the dopamine neurons themselves. By contrast, RNAi against *dop-3* blocked basal slowing in the control strain but did not affect basal slowing in the P*dat-1* strain. We also tested basal slowing following feeding RNAi directed against *unc-13* or *snb-1*. In these experiments, the standard *lin-15B; eri-1* strain could not be tested because knockdown of these genes resulted in an uncoordinated behavioral phenotype (Fig. 2C). By contrast, knockdown of *unc-13* and *snb-1* in the P*dat-1* strain eliminated the basal slowing response (Fig. 3B). Further, although the basal slowing response was eliminated by knockdown of *unc-13* or *snb-1*, these knockdowns did not affect the rate of locomotion (p = 0.0816 and p = 0.5566 respectively, compared to control off food). These data demonstrate that feeding RNAi in the P*dat-1* strain is effective in dopamine neurons but is blocked in other cells.

### Glutamatergic neuron-specific RNAi

Next, we targeted glutamatergic neurons by driving expression of our artificial operon under the P*eat-4* promoter. In *C. elegans*, one behavior mediated by glutamatergic neurotransmission is reversal in response to nose touch [36]. Glutamatergic control of this behavior requires the gene *eat-4*, which encodes VGLUT, the glutamate synaptic vesicle transporter [37] that functions intrinsically in glutamatergic neurons. Glutamate control of the nose touch response also requires the gene *glr-1*, which encodes an AMPA-type ionotropic glutamate receptor [38], [39] and is required in the post-synaptic cells that respond to glutamate. We fed neuron-sensitized (*lin15B; eri-1*) animals dsRNA against *eat-4* and *glr-1*. Under both conditions, these animals were deficient in their ability to respond to nose touch when compared to controls (Fig. 3C). Thus, feeding RNAi can target both pre- and post-synaptic components of glutamatergic neurotransmission to generate a glutamate-specific behavioral defect. We then tested the glutamatergic neuron-specific RNAi strain. On control RNAi, these animals exhibited a normal nose touch response, similar to the standard sensitized strain (Fig. 3C and D, gray bars; p = 0.3421). Feeding RNAi against *eat-4* resulted in a loss of nose touch response compared to empty vector fed controls, similar to the loss in the standard strain (Fig. 3D). Thus, RNAi of an endogenous gene in glutamatergic neurons is effective in the glutamate-specific strain. By contrast, RNAi against *glr-1* did not affect the ability of the glutamate-specific strain to respond to nose touch. This result suggests that unlike the standard sensitized strain, the glutamate-specific strain is insensitive to RNAi outside the glutamate neurons. In support of this, we found that feeding RNAi against the basal neurotransmission genes *unc-13* and *snb-1* resulted in a glutamate-specific behavioral defect in nose touch, rather than a general defect in movement. In addition, glutamate-specific RNAi worms fed dsRNA against *unc-70* were also impaired in their response to nose touch when compared to controls (Fig. 3D), suggesting that *unc-70* is important for maintaining the integrity of glutamate-releasing neurons.

### Cholinergic neuron-specific RNAi

We also targeted cholinergic neurons using the P*unc-17* promoter, which drives expression in acetylcholine-releasing neurons, including the excitatory motor neurons that innervate the body wall muscles and are required for proper locomotion [40], [41]. We first measured the thrashing rate in liquid of neuron-sensitized (*lin-15B; eri-1*) worms and found that they exhibited a significant decrease when fed bacteria producing dsRNA against *snb-1 or unc-13* compared to empty vector control (Fig. 2E). We then knocked these genes down in the acetylcholine-specific RNAi strain and observed a similar decrease in the rate of thrashing (Fig. 2F). We also fed the acetylcholine-specific RNAi worms dsRNA against *unc-70*. These worms were impaired in their thrashing rate but displayed no other phenotypes indicative of systemic RNAi of *unc-70*, suggesting that RNAi in this strain is restricted to the acetylcholine neurons.

### A screen for essential gene function in mature GABA neurons

Neurons are complex cells, and synaptic transmission and maintenance of normal neuronal function requires the concerted action of a large number of genes. Although many such genes have been identified by forward genetic screens, we hypothesized that these screens may have missed important requirements in neurons for essential genes. Essential genes – those required for the growth of an organism to a fertile adult – are difficult to recover in screens for neuronal function because of death, arrest, or sterility of the mutant. Yet such genes might have critical roles in neurons. Accordingly, we sought to expand our ubderstanding of the genetic requirements for proper GABA neuron function by screening our GABA-specific RNAi strain against a large number of essential genes.

**Figure 4:**
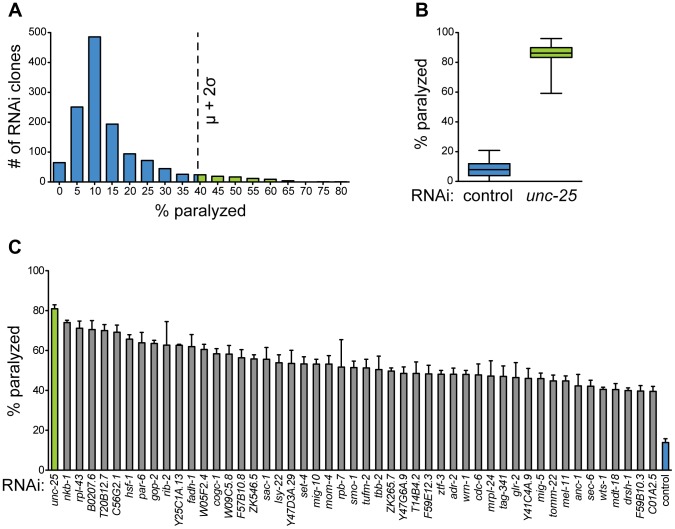
A screen for essential gene function in mature GABA neurons. (**A**) Histogram of primary GABA-specific RNAi screen. 79 RNAi clones (green bars) were selected for retesting with aldicarb hypersensitivity above cutoff (dotted line) of two standard deviations above the mean. μ = 14.40%, σ = 12.15%, cutoff = 38.70%. (**B**) Aldicarb sensitivity of controls from primary screen. Negative control (L4440, blue box) fed worms are significantly less sensitive to aldicarb than positive control (*unc-25*, green box) fed worms. N = 42 for each condition. Whiskers are min to max values. p<0.0001. (**C**) Hits from secondary screen. RNAi against 48 genes causes hypersensitivity to aldicarb above primary RNAi screen cutoff in at least 3 independent trials of ∼25 worms each. p<0.0001 for all clones compared to control RNAi.

We began by curating a list of all essential genes by reported RNAi phenotype of lethal, arrested, or sterile using WormMart (wormbase.org), and we arrayed corresponding Ahringer RNAi [6] clones into a custom essential gene RNAi library. Our primary screen consisted of 1,782 essential RNAi clones from chromosomes I, II, and III. Using the GABA-specific RNAi strain described above, we screened animals fed these clones for hypersensitivity to aldicarb-induced paralysis. Due to the strict experimental control needed for proper neuronal RNAi and phentoyping, such as log-phase culture and age-matched progeny, we were successfully able to screen 1,320 clones (outlined in Table S1) targeting essential genes for their effect on GABA output. From the primary screen, we identified 79 clones (∼6%) that produced aldicarb hypersensitivity of at least two standard deviations above the mean (Figure 4A).

We also examined the morphology of the GABA nervous system in each experiment for branching, degeneration, or cell death. We found that in contrast to the functional defects, we observed no morphological defects in any of the 1,320 RNAi experiments. Thus, the functional deficits we observe in response to RNAi are the result of altered neurotransmission rather than cell death or degeneration. However, our screen was conducted on young adult animals, so it is possible that longer-term knockdown would result in morphological phenotypes for some genes.

To validate the primary screen hits, we retested each of the clones in at least three independent trials. We selected those that retested above the cutoff from the primary screen, which was well above that needed for statistical significance (p<0.0001 for all selected clones compared to control). These clones were then sequenced to confirm the targeted gene. We discarded any clone that could not be mapped to a single gene target, including clones that mapped to more than one gene, intergenic region, or intron. We identified 48 genes (Figure 4C, Table 1) whose knockdown in GABA neurons led to aldicarb hypersensitivity, and thus decreased GABA motor output. Eighty-three percent (40) of these genes have predicted human orthologs [42], suggesting that we have identified a largely conserved set of genes that are important for post-developmental GABA neuron function.

**Table 1:** Essential gene RNAi clones that affect GABA neuron function.

Gene	Chr.	Description	Conserved*	% paralyzed +/− SEM
unc-25	III	glutamic acid decarboxylase (positive control)		80.92%+/−2.07%
nkb-1	I	Na^+^/K^+^ ATPase, beta subunit	YES	74.02%+/−1.15%
rpl-43	II	large ribosomal subunit L37a	YES	71.19%+/−3.59%
B0207.6	I	unknown GTPase	YES	70.51%+/−4.49%
T20B12.7	III	anamorsin homolog	YES	70.05%+/−3.03%
C56G2.1	III	A-kinase anchor protein 1 homolog, mitochondrial	YES	69.23%+/−3.53%
hsf-1	I	HSF1 homolog	YES	65.73%+/−2.24%
par-6	I	PARD6G homolog	YES	63.91%+/−5.21%
gop-2	III	unknown ATP binding protein	YES	63.57%+/−1.57%
rib-2	III	exostosin-like protein	YES	62.67%+/−11.85%
Y25C1A.13	II	ECH1 homolog	YES	62.61%+/−0.55%
fadh-1	III	FAH domain protein	YES	61.93%+/−6.11%
W05F2.4	I	no known domains	NO	60.57%+/−2.59%
cogc-1	I	COG1 homolog	YES	58.39%+/−2.54%
W09C5.8	I	cytochrome c oxidase subunit	YES	58.22%+/−4.28%
F57B10.8	I	activator of basal transcription 1 homolog	YES	56.35%+/−4.13%
ZK546.5	II	zinc finger protein	NO	55.75%+/−2.10%
sac-1	I	SAC1 PIP phosphatase homolog	YES	55.65%+/−5.89%
lsy-22	I	defective in lateral asymmetry 22	NO	53.85%+/−4.02%
Y47D3A.29	III	DNA polymerase alpha catalytic subunit	YES	53.50%+/−6.61%
set-4	II	putative histone H4 lysine-20 methyltransferase	YES	53.33%+/−3.53%
mom-4	I	MAPKKK7 homolog, wnt signaling	YES	53.23%+/−4.19%
mig-10	III	lamellopodin	YES	53.23%+/−2.40%
rpb-7	I	RNA polymerase II subunit	YES	51.72%+/−13.77%
tufm-2	I	elongation factor TU, mitochondrial	NO	51.27%+/−4.37%
tbb-2	III	beta tubulin	YES	50.49%+/−6.70%
ZK265.7	I	no known domains	NO	49.70%+/−1.62%
Y47G6A.9	I	DNA-directed RNA polymerase III subunit	YES	48.58%+/−3.25%
T14B4.2	II	28S ribosomal protein S18c, mitochondrial	YES	48.56%+/−5.76%
F59E12.3	II	no known domains	NO	48.25%+/−4.41%
ztf-3	I	zinc finger transcription factor	NO	48.14%+/−1.86%
adr-2	III	adenosine deaminase	YES	48.11%+/−3.09%
wrn-1	II	WRN homolog	YES	48.00%+/−2.00%
smo-1	I	SUMO1 homolog	YES	47.85%+/−5.63%
cdc-6	I	CDC6 homolog	YES	47.83%+/−5.45%
mrpl-24	II	mitochondrial ribosomal protein, large	YES	47.24%+/−7.62%
tag-341	II	Rho-GAP protein	YES	47.01%+/−5.30%
glr-2	III	AMPA receptor subunit	YES	46.43%+/−7.56%
Y41C4A.9	III	digestive organ expansion factor homolog	YES	46.00%+/−5.03%
mig-5	II	DVL-3 dishevelled homolog, wnt signaling	YES	45.97%+/−2.73%
tomm-22	II	TOMM22 homolog	YES	44.81%+/−2.89%
mel-11	II	myosin-associated phosphatase regulatory subunit homolog	YES	44.81%+/−2.45%
anc-1	I	SYNE1 homolog	YES	42.29%+/−5.78%
sec-6	II	yeast SEC6 homolog	YES	42.15%+/−2.96%
wts-1	I	LATS1 homolog, hippo signaling	YES	40.50%+/−1.12%
mdt-18	I	mediator of RNA polymerase II	YES	40.48%+/−2.99%
drsh-1	I	drosha	YES	39.94%+/−1.39%
F59B10.3	II	no known domains	NO	39.70%+/−2.74%
C01A2.5	I	no known domains	YES	39.53%+/−2.48%
L4440		empty vector control		13.90%+/−1.90%

Chr, chromosome.

* Conservation based on presence on OrthoList [42].

## Discussion

The technique and strains presented here enable cell-specific knockdown in designated neurons simply by performing feeding RNAi. Our results in GABA, dopamine, glutamate, and acetylcholine-releasing neurons suggest that the technique can be used to limit feeding RNAi to any neuron or group of neurons. However, since RDE-1 acts at a rate-limiting step early in the exogenous RNAi pathway, small amounts of misexpression can trigger an amplified interference response – hence, tightly controlled promoters are required to drive expression of the transgene to ensure specificity.

One major use of this technique will be to rapidly determine the site of action of particular genes. For example, our results demonstrate that the dopamine receptor *dop-3* does not function in the dopamine neurons, and the glutamate receptor *glr-1* does not function in the glutamate neurons.

Another major use will be to easily determine the function in specific neurons of genes that are ubiquitously expressed. For example, our data demonstrate that GABA, acetylcholine, dopamine, and glutamate neurons all rely on *unc-13* and *snb-1* for neurotransmission. Also, we show that *unc-70* is required in GABA, acetylcholine, and glutamate neurons for proper function.

Finally, our technique enables new kinds of forward genetic screens, as we have demonstrated with our GABA-specific RNAi screen of essential genes. Essential genes make up a substantial portion of the genes in the genome, but are virtually inaccessible to traditional genetic screens. These genes, however, are some of the most conserved – while only ∼38% of all *C. elegans* genes have human orthologs [42], ∼76% of the genes we selected for their essential RNAi phenotype have predicted orthologs. By using a strain that limits RNAi to non-essential neurons (such as our GABA-specific strain), it is now possible to screen for neuronal functions of genes that normally have lethal, sterile, or other pleiotropic phenotypes.

We have identified 48 genes whose cell-specific knockdown lead to deficits in GABA neurotransmission. As expected, we found components of essential cellular processes such as energy metabolism, transcription, and translation. Additionally, components of several important and conserved signaling and gene regulation pathways were identified, such as miRNA (*drsh-1*), Wnt signaling (*mig-5*), and Hpo signaling (*wts-1*). These pathways have been studied extensively for their role in development, but our data suggest that these pathways may be important post-developmentally for maintenance of GABA neuron function. The genes identified in this study provide a more complete understanding of the complex genetic requirements of post-developmental neurons. Additional studies will be required to determine the mechanism through which these genes act to promote GABA neuron function, whether through specific modulation of neuronal functions such as neurotransmitter release, or general cellular health and metabolism.

The four strains presented here enable the rapid knockdown of many single gene targets in a given neuron sub-type. The efficiency of RNAi in each strain varies, possibly due to differences in expression levels of our bi-cistronic transgene when driven by various promoters. In the case of the GABAergic neuron-specific strain, we are able to recapitulate the null phenotype of *unc-25(e156)* with *unc-25* RNAi (Figure S1A, p = 0.0994). The efficiency of the dopaminergic-specific RNAi strain is comparable to that of the standard sensitized RNAi strain – when fed RNAi against *cat-2* (Figure 3A, B), basal slowing response is abolished to a similar level in both strains. The efficiency of RNAi in the glutamatergic neuron-specific RNAi strain is also comparable to the standard sensitized strain when nose touch behaviors are compared after feeding with *eat-4* RNAi (Figure 3C, D, p = 0.5205). Finally, RNAi in the cholinergic neuron-specific RNAi strain is slightly less efficient than the standard sensitized strain – there is a small, but significant difference in the thrashing rates when these strains are fed RNAi against *unc-13* (Figure 3E, F, p = 0.0430) or *snb-1* (p = 0.0478). Additionally, RNAi of *unc-13* in the cholinergic-specific RNAi strain is unable to fully recapitulate the slowed thrashing rate of *unc-13(e51)* mutants (Figure S1B, p = 0.0003), due to a combination of decreased penetrance and effect size. All the strains presented, however, show dramatic behavioral defects when fed RNAi against genes that are required for those neurons to function.

An interesting feature of our system is that we do not observe effects for knockdown of gene function during development. For example, although GABA neurotransmission is required at all developmental stages for normal movement, our GABA-specific strain does not exhibit behavioral defects until the L4 and adult stage. Similarly, no developmental defects were observed during our GABA-specific screen of essential genes. A likely reason for this is that in our system, RNAi is not initiated until *rde-1* is expressed, and expression from the promoters we use does not begin soon enough to affect behavior at earlier stages. Although this delay means that the developmental functions of genes cannot be studied with our system, it also allows bypassing developmental effects for genes that function both during development and afterward. For example, if a neuronal gene functions in axon guidance and also functions in neurotransmission, our system will allow specific analysis of this later role.

To our knowledge, our approach is the first in any metazoan that enables cell-specific knockdown in any chosen neuron sub-type (with an available promoter), in a way that is high throughput enough to be compatible with questions involving large numbers of genes – including whole-genome screens. As additional specific sensitized strains are developed in addition to the four presented here, it will be possible to combine analysis of neural circuits with genetics, knocking down specific genes in specific parts of circuits and determining the effect on output. In general, we expect this technique will be useful for two major classes of applications: first, to rapidly determine the site of gene function by knocking down a gene of interest in specific neurons; and second, to perform forward genetic screens in a mosaic context, defeating the muddying effects of pleiotropy and biasing the hits toward genes that function intrinsically in the neurons of interest.

## Materials and Methods

### Plasmid construction

All entry clones were generated using Phusion DNA polymerase (Finnzymes) and Gateway BP Clonase II (Life Technologies). *rde-1* and *sid-1* were amplified from genomic and cDNA, respectively, from start to stop codons and cloned into pDONR221 (Life Technologies). The bi-cistronic *rde-1:SL2:sid-1* entry clone was generated using In-Fusion PCR cloning kit (Clonetech) in two steps: first, the 245 bp SL2-specific trans-splice site from the *gpd-2*/*gpd-3* intergenic region [20] was inserted upstream of the start codon of the *sid-1* entry vector, then the *rde-1* gene was inserted upstream of the SL2 site. *Pdat-1* and *Peat-4* promoter entry clones were made by PCR amplification of 717 bp and 2582 bp, respectively, upstream of the corresponding gene start site and cloned into pDONR-P4-P1R. *Punc-47* and *Punc-17* promoter entry constructs [43] were a gift from Gunther Hollopeter, University of Utah, Salt Lake City, UT. Expression clones were generated using Gateway LR clonase II Plus (Life Technologies) and inserted into pCFJ150 [21], a Gateway three-fragment compatible destination vector for MosSCI containing a *C. briggsae unc-119* rescue fragment and genomic regions flanking the *ttTi5605 Mos1* insertion, to generate: pCF1021 (*Punc-47::rde-1:SL2:sid-1::let-858UTR*), pCF1028 (*Punc-17::rde-1:SL2:sid-1::let-858UTR*), pCF1035 (*Pdat-1::rde-1:SL2:sid-1::let-858UTR*), pCF1044 (*Peat-4::rde-1:SL2:sid-1::let-858UTR*). The *unc-70* RNAi construct was made by inserting 1697 bp of *unc-70* coding sequence between the SpeI and KpnI sites of L4440 (clone pPD129.36, Fire Kit, Addgene). Primers and templates are outlined in Table S2.

### Strains and transgenics

All mutant *C. elegans* strains were provided by Caenorhabditis Genetics Center and maintained at 20°C as previously described [44]. Transgenic *C. elegans* lines carrying the transgenes as single copy insertions were created as described [21], [45] using insertion site *ttTi5605*, then verified by PCR and Sanger sequencing. These insertions were then crossed into *lin-15B(n744); eri-1(mg366); rde-1(ne219)* mutant animals and genotyped by PCR, Sanger sequencing, and resistance to *ama-1* RNAi. XE1583 was created by microinjection of XE1375 with 15 ng µl^−1^ of pTG96 [46] as described [47]. The following strains were used in this study: N2, KP3948 (*lin-15B(n744)* X; *eri-1(mg366)* IV), XE1375 (*lin-15B(n744)* X; *eri-1(mg366)* IV; *rde-1(ne219*) V; *wpSi1[Punc-47::rde-1:SL2:sid-1, Cbunc-119(+)]* II; *wpIs36[Punc-47::mCherry]* I), XE1474 (*lin-15B(n744)* X; *eri-1(mg366)* IV; *rde-1(ne219)* V; *wpSi6[Pdat-1::rde-1:SL2:sid-1, Cbunc-119(+)]* II), XE1581 (*lin-15B(n744)* X; *eri-1(mg366)* IV; *rde-1(ne219)* V; *wpSi10[Punc-17::rde-1:SL2:sid-1, Cbunc-119(+)]* II), XE1582 (*lin-15B(n744)* X; *eri-1(mg366)* IV; *rde-1(ne219)* V; *wpSi11[Peat-4::rde-1:SL2:sid-1, Cbunc-119(+)]* II), XE1583 (*lin-15B(n744)* X; *eri-1(mg366)* IV; *rde-1(ne219)* V; *wpSi1[Punc-47::rde-1:SL2:sid-1, Cbunc-119(+)]* II; *wpIs36[Punc-47::mCherry]* I; *wpEx180[Psur-5::sur-5:gfp:NLS]*), CB156 (unc-25(e156) III), MT7929 (unc-13(e51) I).

### RNAi

RNAi was induced by feeding as described [15], with modifications. We found the following conditions were optimal for RNAi in these strains. Standard NGM agar [44] was supplemented with 25 µg ml^−1^ carbenicillin and 1 mM isopropyl β-D-1-thiogalactopyranoside (IPTG), poured into 6 cm dishes or 12-well plates, and allowed to dry for 7 days at room temperature (RT). *E. coli* HT115 carrying the appropriate RNAi clones were grown in LB containing 100 µg ml^−1^ carbenicillin and 50 µg ml^−1^ tetracycline at 37°C overnight. This saturated culture was then seeded 1∶40–1∶200 into LB containing 100 µg ml^−1^ carbenicillin and grown at 37°C until it reached an OD600 of 0.6–0.8, then several drops were seeded onto plates, making sure the culture dried within 1–2 hrs, and induced at RT for 48 hrs. L1 worms were then transferred to the plates (3 per 12-well plate well or 6 per 6 cm plate) and allowed to grow at 20°C for approximately 7 days until young adult (L4+1 day) F1 progeny were visible. The following RNAi clones were used: *L4440* empty vector control (clone pPD129.36, Fire Kit, Addgene), *GFP* (clone pPD128.110, Fire Kit, Addgene), *snb-1* (clone T10H9.4, Ahringer Library [6], *unc-13* (clone ZK524.2, Ahringer Library), *cat-2* (clone B0432.5, Ahringer Library), *dop-3* (clone T14E8.3, Ahringer Library), *eat-4* (clone ZK512.6, Ahringer Library), *glr-1* (clone C06E1.4, Ahringer Library), *unc-25* (clone Y37D8A.23, Vidal ORFeome Library [7], *unc-49* (clone T21C12.1, Vidal ORFeome Library). See Supplemental Table S1 for the list of clones used in the screen.

### Microscopy

Young adult hermaphrodites were mounted in a slurry of 0.1 µm diameter polystyrene beads (Polysciences) on a 5% agarose pad and imaged using a UltraVIEW VoX (Perkin Elmer) spinning disc confocal microscope using a 60× CFI Plan Apo VC, NA 1.4, oil objective. Images were pseudo colored in Fiji [48].

### Behavioral assays

Aldicarb hypersensitivity was measured as described [28]. Briefly, NGM agar was poured into 12-well plates and allowed to dry for 14 days at RT. Plates were then weighed, top-spread with 30 mM aldicarb (Ultra Scientific) to a final concentration of 750 µM, and allowed to dry for 6 hrs. Plates were then seeded with 5 µl of OP50 culture and allowed to grow overnight at RT. 25 young adult worms were then transferred to each well, and after 100 min, the number of paralyzed (defined as the cessation of all spontaneous movement) worms was counted. Each experiment was performed in triplicate.

Basal slowing was measured as described [33]. Locomotion for 20 worms was measured for each RNAi and each treatment.

Thrashing was measured by picking a single young adult worm into a drop of M9 buffer [44] on a glass slide at RT. After equilibrating for 30 sec, the number of body bends (complete movement of the anterior of the worm from one extreme to the other and back) was counted for 30 sec. Rates were measured for 10 worms for each RNAi treatment.

Response to nose touch was measured as described [36]. 10 animals were tested for each condition, 5 trials/animal. The percentage of reversals per total trials was calculated.

### Screen

RNAi was performed as described above, with cultures grown in 96-deep-well plates. Two positive (*unc-25*) and two negative (*L4440*) controls were included for each 96 clones screened. Only cultures grown to log-phase were seeded onto plates as measured by OD600 using a Perkin Elmer Victor 2 plate reader. Aldicarb hypersensitivity was performed as above. Morphology of GABA neurons was examined using a Leica M165FC epi-fluorescent dissecting microscope under 500× magnification.

### Statistical analysis

Two-tailed, unpaired Student's t-tests were used to compare GFP RNAi and aldicarb hypersensitivity data in Figures 2 and 4, as well as basal slowing and thrashing in Figure 3. Two-tailed Fisher's exact test was used to compare nose touch behaviors in Figure 3.

## Supporting Information

Figure S1 A comparison of neuron-specific RNAi phenotypes to mutants. (**A**) Aldicarb sensitivity of the GABAergic neuron-specific RNAi strain (grey bars) and isogenic strains (black bars). *unc-25* RNAi recapitulates the null phenotype of *unc-25(e156)*. Error bars are SEM, n = 3 trials for RNAi, 5 trials for isogenic strains of ∼25 animals each (**B**) Thrashing rates of the cholinergic neuron-specific RNAi strain (closed circles) and isogenic strains (open circles). Each circle represents an individual worm, line is mean, error bars are SEM, n = 10 worms.
(TIF)

Table S1 Essential genes screened in primary GABAergic neuron-specific RNAi screen. 1,320 essential gene RNAi clones and the resulting aldicarb sensitivity when fed to the GABAergic neuron-specific RNAi strain.
(XLS)

Table S2 Primers and templates used for plasmid construction.
(DOC)
