# Supplementary figures and images for: Neuron-Specific Feeding RNAi in C. elegans and Its Use in a Screen for Essential Genes Required for GABA Neuron Function
Source: PLoS Genet. 2013 Nov 7;9(11):e1003921. doi: 10.1371/journal.pgen.1003921 (PMC3820814; doi:10.1371/journal.pgen.1003921)

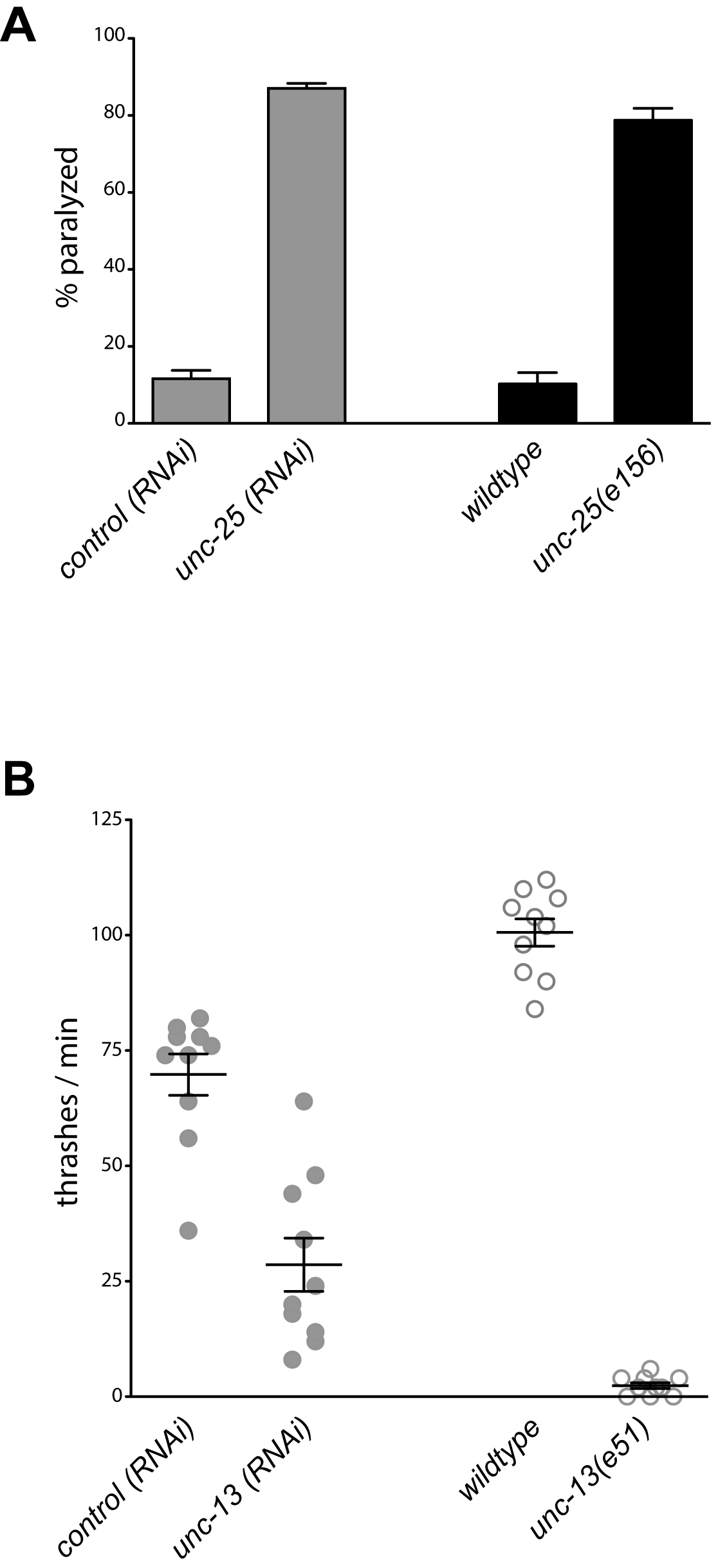

Supplement: Figure S1 — A comparison of neuron-specific RNAi phenotypes to mutants. (A) Aldicarb sensitivity of the GABAergic neuron-specific RNAi strain (grey bars) and isogenic strains (black bars). unc-25 RNAi recapitulates the null phenotype of unc-25(e156). Error bars are SEM, n = 3 trials for RNAi, 5 trials for isogenic strains of ∼25 animals each (B) Thrashing rates of the cholinergic neuron-specific RNAi strain (closed circles) and isogenic strains (open circles). Each circle represents an individual worm, line is mean, error bars are SEM, n = 10 worms. (TIF) [file pgen.1003921.s001.tif]
