# Supplementary material for: Neuron-Specific Feeding RNAi in C. elegans and Its Use in a Screen for Essential Genes Required for GABA Neuron Function
Source: PLoS Genet. 2013 Nov 7;9(11):e1003921. doi: 10.1371/journal.pgen.1003921 (PMC3820814; doi:10.1371/journal.pgen.1003921)
Supplement: Table S2 — Primers and templates used for plasmid construction. (DOC) [file pgen.1003921.s003.doc]

Supplementary Table 2: Primer sequences

| Primer Name | Sequence | Template |
| --- | --- | --- |
| **rde-1[1-2] entry 5’** | ggggacaagtttgtacaaaaaagcaggctaaaaATGTCCTCGAATTTTCCCGAATTGGAAAAAG | N2 genomic |
| **rde-1 [1-2] entry 3’** | ggggaccactttgtacaagaaagctgggtTTATGCGAACGACATTCCAGGGTAC |  |
| **sid-1 [1-2] entry 5’** | ggggacaagtttgtacaaaaaagcaggctATGATTCGTGTTTATTTGATAATTTTAATGCATTTGGTG | N2 cDNA |
| **sid-1 [1-2] entry 3’** | ggggaccactttgtacaagaaagctgggtCTAGAAAATGTTAATCGAAGTTTTGCGTGTATTAATGAG |  |
| **sid-1 InF open 5'** | ATGATTCGTGTTTATTTGATAATTTTAATGCATTTGGTG | *sid-1* [1-2] entry |
| **sid-1 InF open 3'** | AGCCTGCTTTTTTGTACAAAGTTGG |  |
| **SL2 InF sid-1 5'** | acaaaaaagcaggctGCTGTCTCATCCTACTTTCACCTAGTTAAC | N2 genomic |
| **SL2 InF sid-1 3'** | ataaacacgaatcatGATGCGTTGAAGCAGTTTCCCTG |  |
| **SL2:sid-1 InF open 5'** | GCTGTCTCATCCTACTTTCACCTAG | *SL2:sid-1* [1-2] entry |
| **SL2:sid-1 InF open 3'** | AGCCTGCTTTTTTGTACAAAGTTGG |  |
| **rde-1 InF SL2:sid-1 5'** | acaaaaaagcaggctATGTCCTCGAATTTTCCCGAATTGG | *rde-1* [1-2] entry |
| **rde-1 InF SL2:sid-1 3'** | gtaggatgagacagcTTATGCGAACGACATTCCAGGG |  |
| **Pdat-1 [4-1] entry 5'** | ggggacaactttgtatagaaaagttgTCCATGAAATGGAACTTGAATCCAGTTTTC | N2 genomic |
| **Pdat-1 [4-1] entry 3'** | ggggactgcttttttgtacaaacttgGGCTAAAAATTGTTGAGATTCGAGTAAACCG |  |
| **Peat-4 [4-1] entry 5'** | ggggacaactttgtatagaaaagttgCTGCAGCAATGATCGAACTTTCTC | N2 genomic |
| **Peat-4 [4-1] entry 3'** | ggggactgcttttttgtacaaacttgGGTTTCTGAAAATGATGATGATGATGATGGAG |  |
| **unc-70 RNAi 3’** | catgactagtTCGATTGGAGTTGTCAATGG | N2 genomic |
| **unc-70 RNAi 5’** | catgggtaccGTCTCTGGGTGGCAGTAAGC |  |
